# The ‘W’ Prawn-Trawl with Emphasised Drag-Force Transfer to Its Centre Line to Reduce Overall System Drag

**DOI:** 10.1371/journal.pone.0119622

**Published:** 2015-03-09

**Authors:** Cheslav Balash, David Sterling, Jonathan Binns, Giles Thomas, Neil Bose

**Affiliations:** 1 National Centre for Maritime Engineering & Hydrodynamics—Australian Maritime College, University of Tasmania, Launceston, Tasmania, Australia; 2 Sterling Trawl Gear Services, Brisbane, Queensland, Australia; University of California San Diego, UNITED STATES

## Abstract

For prawn trawling systems, drag reduction is a high priority as the trawling process is energy intensive. Large benefits have occurred through the use of multiple-net rigs and thin twine in the netting. An additional positive effect of these successful twine-area reduction strategies is the reduced amount of otter board area required to spread the trawl systems, which leads to further drag reduction. The present work investigated the potential of redirecting the drag-strain within a prawn trawl away from the wings and the otter boards to the centre line of the trawl, where top and bottom tongues have been installed, with an aim to minimise the loading/size of the otter boards required to spread the trawl. In the system containing the new ‘W’ trawl, the drag redirected to the centre-line tongues is transferred forward through a connected sled and towing wires to the trawler. To establish the extent of drag redirection to the centre-line tongues and the relative drag benefits of the new trawl system, conventional and ‘W’ trawls of 3.65 m headline length were tested firstly over a range of spread ratios in the flume tank, and subsequently at optimum spread ratio in the field. The developed ‘W’ trawl effectively directed 64% of netting-drag off the wings and onto the centre tongues, which resulted in drag savings in the field of ∼20% for the associated ‘W’ trawl/otter-board/sled system compared to the traditional trawl/otter-board arrangement in a single trawl or twin rig configuration. Furthermore, based on previously published data, the new trawl when used in a twin rig system is expected to provide approximately 12% drag reduction compared to quad rig. The twin ‘W’ trawl system also has benefits over quad rig in that a reduced number of cod-end/By-catch Reduction Device units need to be installed and attended each tow.

## Introduction

Successful drag reduction for trawl systems has primarily occurred through twine area reduction, either from removing netting; or incorporating larger size mesh, thinner twine, knotless netting; or a combination of these. For example, a 18% drag reduction occurs for bottom trawls used by the Portuguese fleet through applying larger mesh netting in the wings and steeper tapers in the wings and bellies [1]. Similarly, drag reduction is achieved by employing high breaking strength netting, which allows the use of thinner twine. For example, in Australian prawn trawls the drag reduction of a trawl constructed with high-strength netting was measured to be 22% when matched with smaller otter boards [2]. Furthermore, knots produce an amplified effect on the overall drag of a prawn trawl compared to their contribution to twine area [3]. This effect was hypothesised to be due to the added in-plane roughness of knotted netting sheets, given that prawn trawls have a large proportion of netting orientated nearly parallel to the flow.

**Fig 1:**
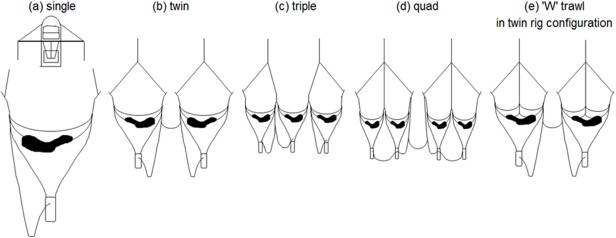
Multiple net configurations: engineering efficiency is progressively increased with an addition of smaller size nets. The proposed ‘W’ trawl in twin rig configuration (e) is hypothesised to be a progressive development beyond the quad rig (d).

In prawn trawling practise, significant drag reduction is obtained through use of multiple-net rigs, which replace a single net with a number of smaller-size nets. The drag reduction occurs because: (1) less netting is used in the system, (2) smaller otter boards are required to spread the smaller nets, and (3) drag savings occur when nets are joined together due to reduced number of otter boards per trawl. Fig. 1 includes a comparative display of some common prawn-trawl systems used in Australia: single (a), twin (b), triple (c) and quad (d) configurations. A simplistic prediction of drag reduction from using multiple nets based on flume tank drag data for modelled Australian prawn trawls of various sizes indicated that triple- and quad-rig trawl systems have less than half the drag of a single-net system with the same total headline length [4]. In the field, based on the measured drag of single, twin, triple and quad rigs, fuel savings of up to 26% were demonstrated from using the high-order multiple net rigs [5]. It should be noted, however, that a negative side-effect of employing multiple nets is the added operational difficulty of emptying multiple cod-ends each tow and the high cost to purchase and maintain a larger number of approved By-catch Reduction Devices (BRD) and Turtle Exclusion Devices (TED).

Looking forward, the authors of the present paper investigate a new technology for further drag reduction of prawn trawl systems, called the ‘W’ trawl [6]. The principle behind the proposal is that a large proportion of netting drag can be transferred to a central towing wire via a trawl sled connected to upper and lower tongues, as opposed to putting load on the otter boards through the wings of the trawl (Fig. 1E) [4]. The new technology is predicted to provide superior drag performance compared to a single trawl configuration due to reduced otter board size requirements, and similarly two ‘W’ trawls could be used instead of standard twin rig for the same performance benefits. Furthermore, the ‘W’ trawl concept would alleviate the operational issue of additional cod-ends, TEDs and BRDs intrinsic to four net systems (quad rig) if the two adjacent nets were replaced by ‘W’ trawls. Potentially the twin ‘W’ trawl system could out-perform quad rig from an engineering perspective, depending on the extent that the ‘W’ trawl can redirect netting-drag to the centre bridle.

Alternation of mesh pattern (diamond—T0 and square—T45) in the upper/lower and side sections of standard and 'W' trawl models did not substantially affect their drag [6]. However, for the ‘W’ trawl, T45 mesh in the upper/lower sections of the trawl was shown to produce a higher transfer of netting-drag force forward to the tongues compared to T0 mesh (40% for T0 and 59% for T45). Conversely, T0 mesh provided an overall favourable solution for the following two reasons: (1) drag transfer to the tongues for the T0 case could be increased through installing bracing ropes down the centre lines of the upper and lower panels of the trawl and (2) the ‘W’ trawl will be significantly cheaper with T0 mesh in the upper/lower panels because standard knotted trawl netting can be used, as opposed to T45 orientation where significant asymmetric (distorted) trawl-shape occurs, unless expensive knotless netting is used [6]. Additionally, it is likely that insufficient netting strength along the bars is available from knotted material when used in this T45 application and expensive Ultracross netting would be required for at least for the highly loaded central sections, given the results of netting-strength tests [4].

The present work investigated the potential of further drag transfer to the tongues through variant bracing rope techniques applied to the centre line of the ‘W’ trawl. Standard (Florida flyer) and ‘W’ trawls of 3.65 m (2 fathom) headline length were tested in the flume tank over a range of spread ratios to compare their engineering performance and monitor performance improvements for the ‘W’ trawl as refinements to the design were implemented. Following the flume tank sessions, the trawls were tested at sea to establish their relative engineering and catching performance in a commercial fishing situation.

## Methods

### Models

**Fig 2:**
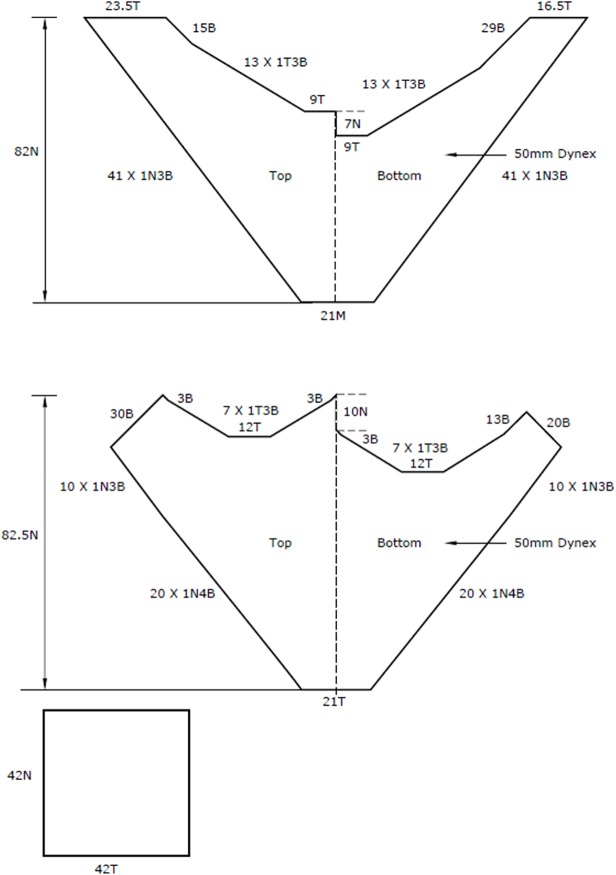
Net plans for Florida flyer (top) and ‘W’ trawl (bottom).

Net plans for the standard and ‘W’ trawls are shown in Fig. 2. The standard trawl was a Florida flyer design typically used by Australian fishermen. Trawls of this 2 fathom size are commonly used as try-gear, a small net to regularly sample catch rates while the main gear is operated uninterrupted over a longer time period. Both trawls were built from Dynex material −50 mm mesh size and 1.1 mm twine thickness.

### Flume tank experiments

The work in the flume tank aimed to:

- Improve the drag transfer to the tongues through implementation of an optimal bracing rope technology along the upper and lower centre lines of the body section.
- Obtain drag and in-pull measurements for the standard and ‘W’ trawls over a range of spread ratios so that the drag benefits of the new system and optimal otter board size could be estimated.

**Fig 3:**
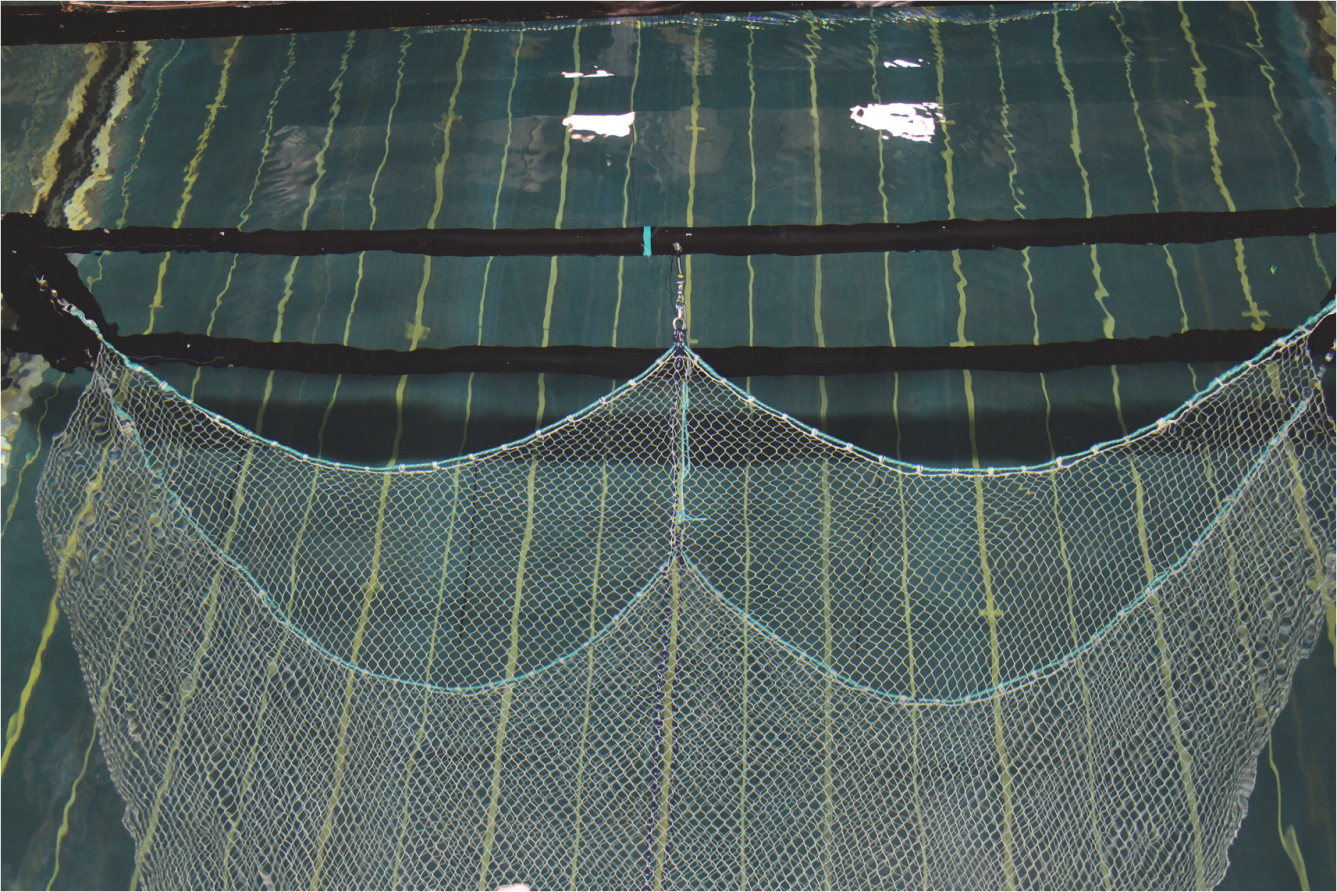
‘W’ trawl attached to the Trawl Evaluation Rig (TER) and placed in the mid-stream section of the flume tank.

The experiments were conducted in the flume tank at the Australian Maritime College—University of Tasmania in Beauty Point, Tasmania, Australia, using trawls constructed of full-scale netting. The trawls were attached one at a time to an aluminium rectangular frame (Trawl Evaluation Rig—TER) by the four end points of the upper and lower frame lines, and an additional two points for the ‘W’ trawl, being at the upper and lower tongues (Fig. 3). Each trawl-connection point contained a load cell so that the frame-line tensions at all connection points were measured for each case. Otter boards and ground chain were not attached to the trawls in these flume tank sessions. The spread ratio was adjusted on the TER by moving the two vertical sides to the desired location and firmly fixing them in place. The tensions measured with 20 kgf capacity load cells at the tow points of the model were recorded for 60 seconds at 5 Hz. To isolate the orthogonal drag and in-pull components from the frame line tensions, the divergence angles of the frame-lines (at the connection points) relative to the flow direction were estimated from repeated manual measurements using a bevel gauge referenced to a transverse beam of the TER, and then the forces were resolved using right-angle triangle trigonometry.

**Fig 4:**
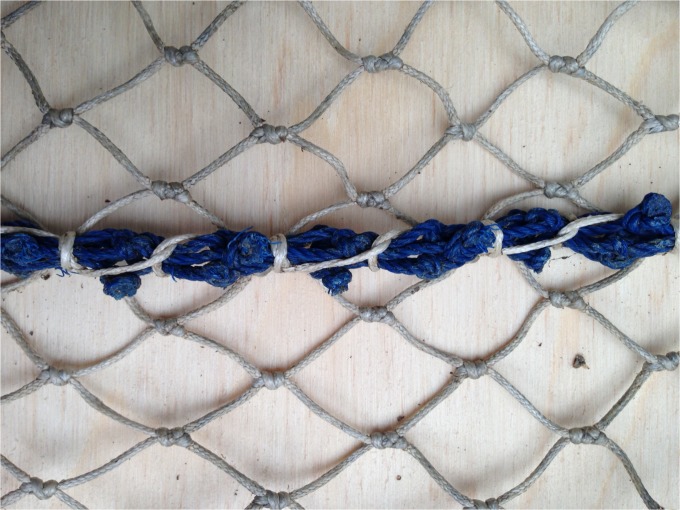
Stretched netting sewn down the centre line with a hanging ratio of 0.707

To investigate the proportion of trawl-drag transferred to the tongues, the following two applications of bracing ropes to the centre line of the trawl were tested with the trawl spread set to 80% of its headline length:

- 3 x 12 mm polyethylene (PE) ropes fitted down the centre line; these ropes were not sewn to the trawl, but were threaded through adjacent meshes to completely fill the open mesh and therefore prevent them from closing.
- Stretched netting was sewn down the centre line of the trawl. The mesh size for the stretched netting was selected so that the position of each knot corresponded to the position of the connection-lashings to the trawl such that a hanging ratio of 0.707 was achieved (Fig. 4).

The optimal bracing rope solution was selected based on the highest drag transfer achieved without distorting the operational shape of the trawl. The selected solution was implemented for a subsequent set of comparative tests between the standard and ‘W’ trawls over spread ratios of 70, 80 and 90% and four flow speeds as specified in Table 1 and 2.

**Table 1:** Tested conditions for flume tank sessions.

Bracing rope application	Trawl spread ratio [%]
3 x 12 mm PE ropes	80
Stretched netting	80
Optimal solution	70
	80
	90

**Table 2:** Flow speeds tested.

Tank impellers rotation [rpm]	Approximate flow speed [m s^−1^]
120	0.96
140	1.12
160	1.28
180	1.44

### Field experiments

The objective of the field experiments was to obtain comparative system drag and spread measurements, and catch data, for a preliminary assessment of the energy efficiency of the new ‘W’ trawl system compared to the standard arrangement.

The trials occurred in Moreton Bay, Queensland, Australia (27.20 south latitude and 153.20 longitude) in September 2013. The experiments occurred before the main prawn season, but this was not a point of concern as catches would be sufficient to gauge the relative effectiveness of the gear. Data collection in respect to catch involved weighing the various catch components during the normal flow of material in the production process. To mitigate the risk of impacts on species of conservation interest, fishing operations in Moreton Bay are subject to a wide range of regulations in relation to the fishing gear used and the location and timing that fishing occurs. The research trials were undertaken in accordance with these regulations. During the trials there were no interactions with any species of conservation interest. No specific permit was required from the Moreton Bay Marine Park because the experiments were conducted within a "blue" zone where commercial trawling and associated trials are permitted. No permit was required from the Fisheries section of Queensland Department of Agriculture, Fishery and Forestry because the gear being used was compliant with all commercial fishing regulations and the trawling was conducted at locations open to commercial fishing at that time.

**Fig 5:**
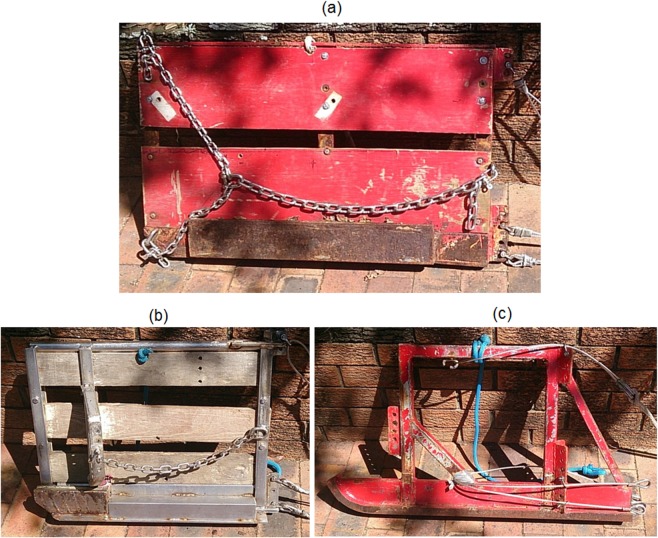
Otter boards and sled used with the small nets; (a) Florida flyer board, (b) ‘W’ trawl board, (c) ‘W’ trawl sled.

The two trawl systems were towed simultaneously in a twin rig configuration: The Florida flyer was positioned on the starboard side of the vessel and the ‘W’ trawl on the port side. 18 tows were undertaken in water depths between 7.7 and 16.1 m over four days and the duration of each tow was 20 min. The last two tows were excluded from the analysis of engineering performance due to failure of the spread sensors. A load cell of 4 tonne capacity was attached using wire grips to the warp on each side of the vessel after the gear was deployed. The towing speed was approximately 2.8 knots. Scanmar sensors were attached on the end points of the upper frame lines to record the horizontal spread of the trawls. The tows were conducted on FV Remark, a 12 m long commercial trawler with 180 HP engine. General particulars for otter boards and sled are presented in Fig. 5 and Table 3. For each tow the amount of warp deployed was recorded along with trawl direction. During each tow regular records were manually taken for warp tension and spread for the port and starboard sides, GPS speed, and water depth. The latter data were measured using the instrumentation fitted to FV Remark and was used to estimate the drag component of each warp-tension measurement and standardise the drag and spread data for the effects of trawl speed and warp declination.

**Table 3:** Otter board and sled particulars.

	Overall length x height dimensions [mm]	Effective panel area [m2]	Weight [kg]
Florida flyer board	914 x 559	0.464	41
‘W’ trawl board	610 X 483	0.232	17
‘W’ trawl sled	914 x 483	in line with flow	34

### Data Analysis

For each flume tank run, 300 recorded tension measurements from each load cell were averaged. Netting drag, *F*_*d*,_ and trawl in-pull, *F*_*in*_, were estimated from summation of averaged tension from each of the six load cells, *T*_*n*_, resolved into the appropriate orthogonal component by the average measured divergence angle for the corresponding frame-line, *θ*_*n*_, as shown in Eq. (1) and (2):

$$F_{d}=\sum_{n=1}^{6}\left(T_{n}\;\cos\theta_{n}\right)$$ (1)

$$F_{in}=\sum_{n=1}^{2}T_{n}\;\sin\theta_{n}\;,\;\sum_{n=3}^{4}T_{n}\;\sin\theta_{n}$$ (2)

For *F*_*in*_, two independent measures were produced for each tank run; one from each side of the trawl (frame-lines 1 & 2 and frame-lines 3 & 4). These were pooled in the subsequent analysis described below.

In the flume tank tests, dynamic non-uniform speed and variation in the measured frame-line divergence angles caused uncertainty in the indicated trawl forces (drag and in-pull). Linear models (with intercept set to zero) were fitted to each set of forces vs. impeller rpm squared, and an estimated mean force was obtained corresponding to the tank flow setting of 180 rpm (∼1.44 m s^-1^). 95% confidence intervals were constructed for each mean force based on the statistical results of the linear fit.

From these performance indicators for the tested trawls, the total drag of the trawl system (including predictions of otter board drag) was estimated from Eq. (3).

$$\mathrm{Total Drag}=\;F_{d}+2\frac{F_{in}}{L/D}$$ (3)

where *L/D* is a lift-to-drag ratio of the otter board, which was assumed to be 1.

Total drag was standardised by spread ratio to indicate the drag per unit of swept area, and plotted against spread ratio to identify the optimum spread ratio for each trawl system. A formal estimation of the optimum spread ratio was achieved by fitting a parabola through the standardised drag versus spread ratio result and calculating the location of minimum standardised drag from the respective quadratic parameters.

The ultimate indication of the benefit of the ‘W’ trawl versus the Florida flyer was the estimated ratio of standardised drag for each trawl system while at its optimum spread ratio. A 95% confidence interval for this measure was obtained using the rules for propagation of variance given in Eq. (4) and (5):

$$y=f\;\left(x_{1},x_{2}\right)\;\sigma_{y}^{2}={\left(\frac{\delta f}{\delta x_{1}}\right)}^{2}\sigma_{x_{1}}^{2}+{\left(\frac{\delta f}{\delta x_{2}}\right)}^{2}\sigma_{x_{2}}^{2}+\;2\frac{\delta f}{\delta x_{1}}\frac{\delta f}{\delta x_{2}}\sigma_{x1}\sigma_{x2}$$ (4)

$$\{\begin{array}{l} y_{1}=f\left(x_{1},x_{2}\right)\\ y_{2}=f\left(x_{1},x_{2}\right) \end{array}\;\sigma_{y_{1}y_{2}}=\frac{\delta f_{1}}{\delta x_{1}}\frac{\delta f_{2}}{\delta x_{1}}\sigma_{x_{1}}^{2}+\frac{\delta f_{1}}{\delta x_{2}}\frac{\delta f_{2}}{\delta x_{2}}\sigma_{x_{2}}^{2}+\left(\frac{\delta f_{1}}{\delta x_{1}}\frac{\delta f_{2}}{\delta x_{1}}+\frac{\delta f_{1}}{\delta x_{2}}\frac{\delta f_{2}}{\delta x_{2}}\right)\sigma_{x_{1}x_{2}}$$ (5)

For the field data, warp tension measurements were converted to estimates of drag by correcting for declination angle at the gear and the buoyed-weight of the wires (0.222 kg m^−1^) used in each haul to tow the gear. A correction for these effects was achieved by fitting a catenary shape to the known characteristics of the towing wires: tension at the boat, length, depth and weight per unit length. A Microsoft Excel macro was used to step through each of the data records and use solver to search for a catenary that fitted the associated data. Once fitted the estimated gear drag for that scenario was added to the data record.

Estimated marginal means for drag, spread, catch of target species (by number and weight) and by-catch weight were obtained for each tested trawl systems by forming a Generalised Linear Model (GLM), using SPSS, of the data from all field tows. The GLM had trawl system and tow as fixed affects for all models, and included trawl speed and the declination of the warp (ratio of deployed warp length (including bridles) to water depth) as covariates for the drag and spread models. The estimated means were then statistically compared on the basis of testing the null-hypothesis that trawl system type had no effect on the dependent variable.

Using the same approach as the flume tank trials, total drag was standardised by spread ratio to indicate the drag per unit of swept area, and the ultimate indication of the benefit of the ‘W’ trawl versus the Florida flyer was the estimated ratio of standardised drag for each trawl system with 95% confidence interval for this measure obtained using the rules for propagation of variance given in Eq. (4) and (5).

## Results

### Engineering performance in the flume tank

For the case of fitting 3 x 12 mm PE ropes down the centre line of the ‘W’ trawl, a large drag-transfer of 72% to the tongues was recorded for 80% spread. However, it was found that the rope fitting process did not easily give a consistent and desired restriction of mesh elongation, and caused excessive trawl-shape distortion. A significant practical improvement was achieved with the subsequent lashed attachment of stretched netting down the centre lines of the upper and lower panels. A 64% drag transfer to the tongues was obtained, while the shape of the trawl was smooth and undistorted.

**Fig 6:**
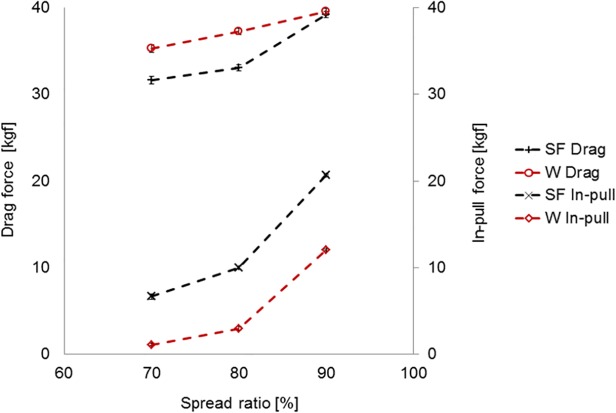
Drag and in-pull with 95% confidence intervals for standard flyer (SF) and ‘W’ trawls with respect to spread ratio.

**Fig 7:**
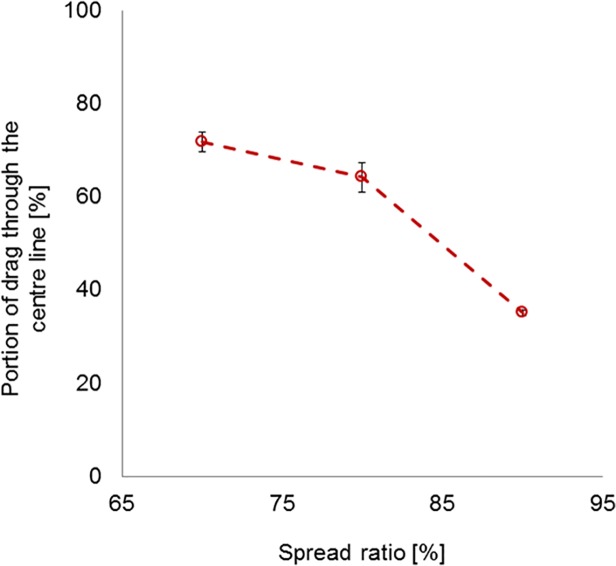
The proportion of drag-transfer through the centre line of the ‘W’ trawl with 95% confidence intervals versus spread ratio.

Fig. 6 shows the estimated performance indicators from the trawl evaluation rig in the flume tank for the two trawls over a range of spread ratios—i.e. indicative drag and in-pull resolved from the load cell tensions recorded over a range of flow speeds for the standard flyer (SF) and ‘W’ trawl. 95% confidence intervals plotted for each data point indicate that there are generally highly significant differences in performance. The ‘W’ trawl typically exhibited a higher drag compared to the standard trawl. However, the difference reduced as spread ratio increased and there was no significant difference (p < 0.05) at the highest spread ratio (90%). Conversely, the ‘W’ trawl in-pull force that would be applied to the otter boards was substantially less than that for the standard trawl (by ∼70% for 80% spread ratio), which was due to the drag-transfer to the tongues and away from the wings of the trawl. As shown in Fig. 7, the proportion of drag-transfer to the tongues decreased as the trawl spread became wider.

**Fig 8:**
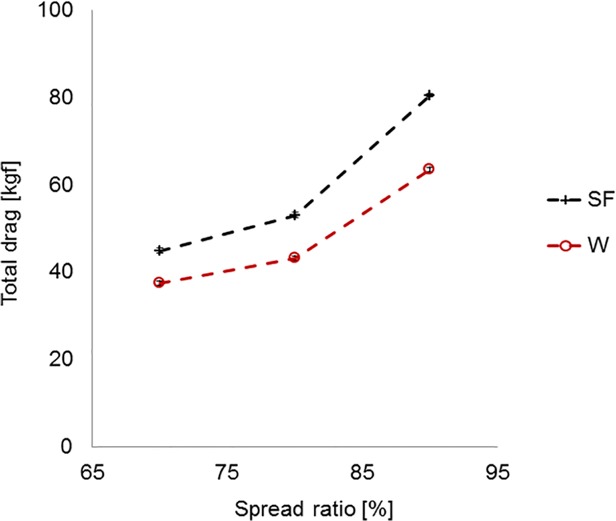
Predictions of total drag for standard flyer (SF) and ‘W’ trawl systems, with 95% confidence intervals [from flume tank data].

Fig. 8 shows predictions of the total drag (trawl and otter board drag) for the three tested spread ratios, along with 95% confidence intervals—the drag difference between the trawl systems was highly significant (p < 0.05) and increased from 16.8% to 20.9% for the spread ratio range of 70% to 90%.

**Fig 9:**
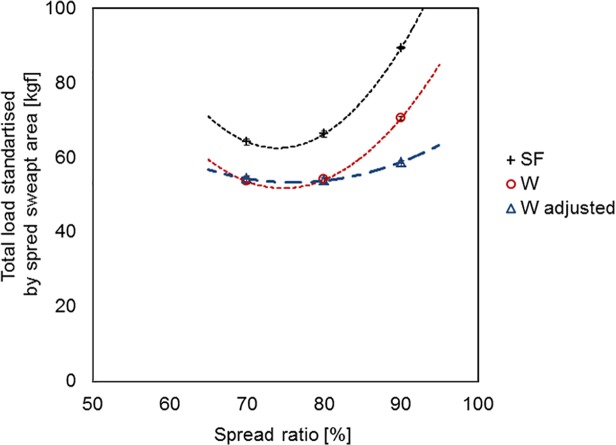
Total drag standardised by swept area rate for standard flyer (SF) and ‘W’ trawl system [from flume tank data].

Based on the flume tank results, the optimal spread ratio, where the drag per unit of spread is minimum, appears to be around 75% for both trawl systems (Fig. 9). This is surprising as one would expect the optimum spread ratio (SR) for the ‘W’ trawl system to be higher than for the standard trawl because the ‘W’ trawl requires smaller otter boards. However, we can see that the reason for the premature minimum in drag/unit-spread for the ‘W’ trawl is because of the way that the proportion of trawl drag transferred to the tongues drops steeply with increasing spread ratio. With hindsight we now understand that this apparent characteristic of the ‘W’ trawl data is an artefact of the methodology used in that the for/aft position of the tongue relative to the wings was set for 80% SR and was not subsequently readjusted appropriately for the 70% and 90% cases. The implication of this situation is that the relative performance result for the ‘W’ trawl at 80% is correct, but the results across the SR range are distorted. A correction can be applied to the results by assuming that the proportion of trawl drag through the centre line would be fixed at 64.2% if the tongue position was correctly adjusted for each SR, rather than drop from 71.9% at 70% SR to only 35.0% at 90% SR. Fig. 9 shows adjusted standardised drag results for the “W” trawl system, which indicates that the practical optimum spread ratio for the ‘W’ trawl has shifted towards 80% and is higher than for the standard trawl (∼75%). When both systems are operating optimally the benefit of the ‘W’ trawl system is estimated to be 14.5 ± 0.51% reduction in standardised drag (drag per unit of spread).

### Engineering and catch performance at sea

**Fig 10:**
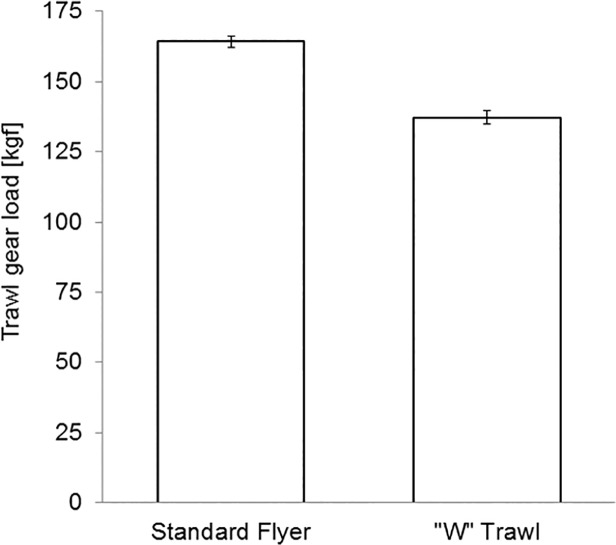
Total drag for standard flyer and ‘W’ trawl systems with 95% confidence intervals [field results].

**Fig 11:**
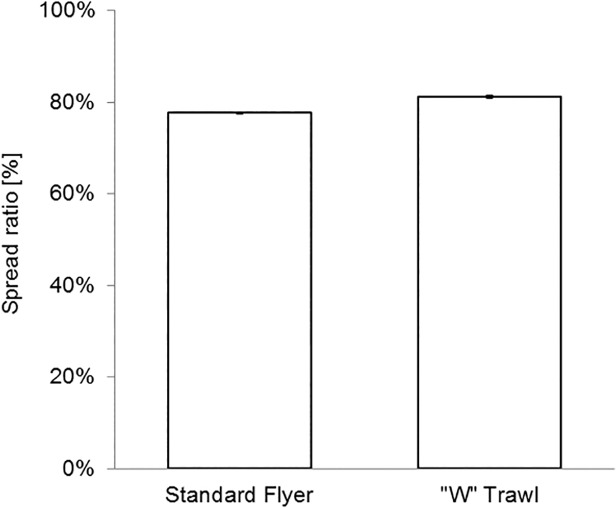
Estimated mean spread ratio with 95% confidence error bars [field results].

Fig. 10 and 11 present the estimated marginal means of total drag and spread ratio for the standard flyer and ‘W’ trawl systems in the field. The ‘W’ trawl had 16.4% less drag, which was statistically significant (p < 0.05), while the predicted mean spread ratio was 81% for the ‘W’ trawl and 78% for the Florida flyer. Table 4 summarises the relative engineering performance of the ‘W’ trawl and shows that the new system provides 20.0 ± 0.7% drag saving when standardised by swept area rate.

**Table 4:** Performance of the ‘W’ trawl relative to the standard system, concluding with standardised drag, and 95% confidence intervals.

Trawl	Drag	Spread ratio	Standardised Drag
Standard	1	1	1
‘W’	0.836 ± 0.005	1.045 ± 0.006	0.800 ± 0.007

**Fig 12:**
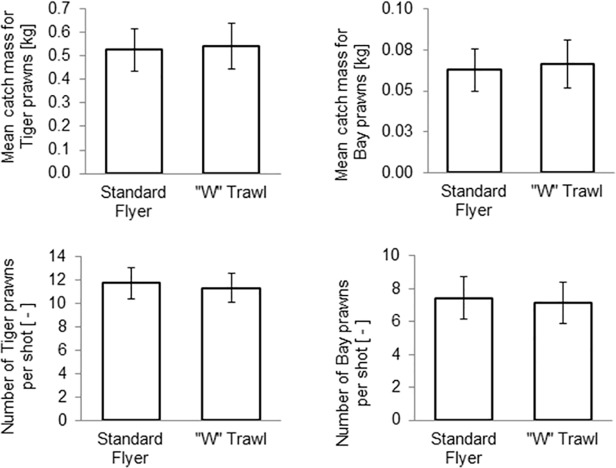
Predicted mean catches per tow by mass and numbers for Tiger and Bay prawns, with 95% confidence intervals.

**Fig 13:**
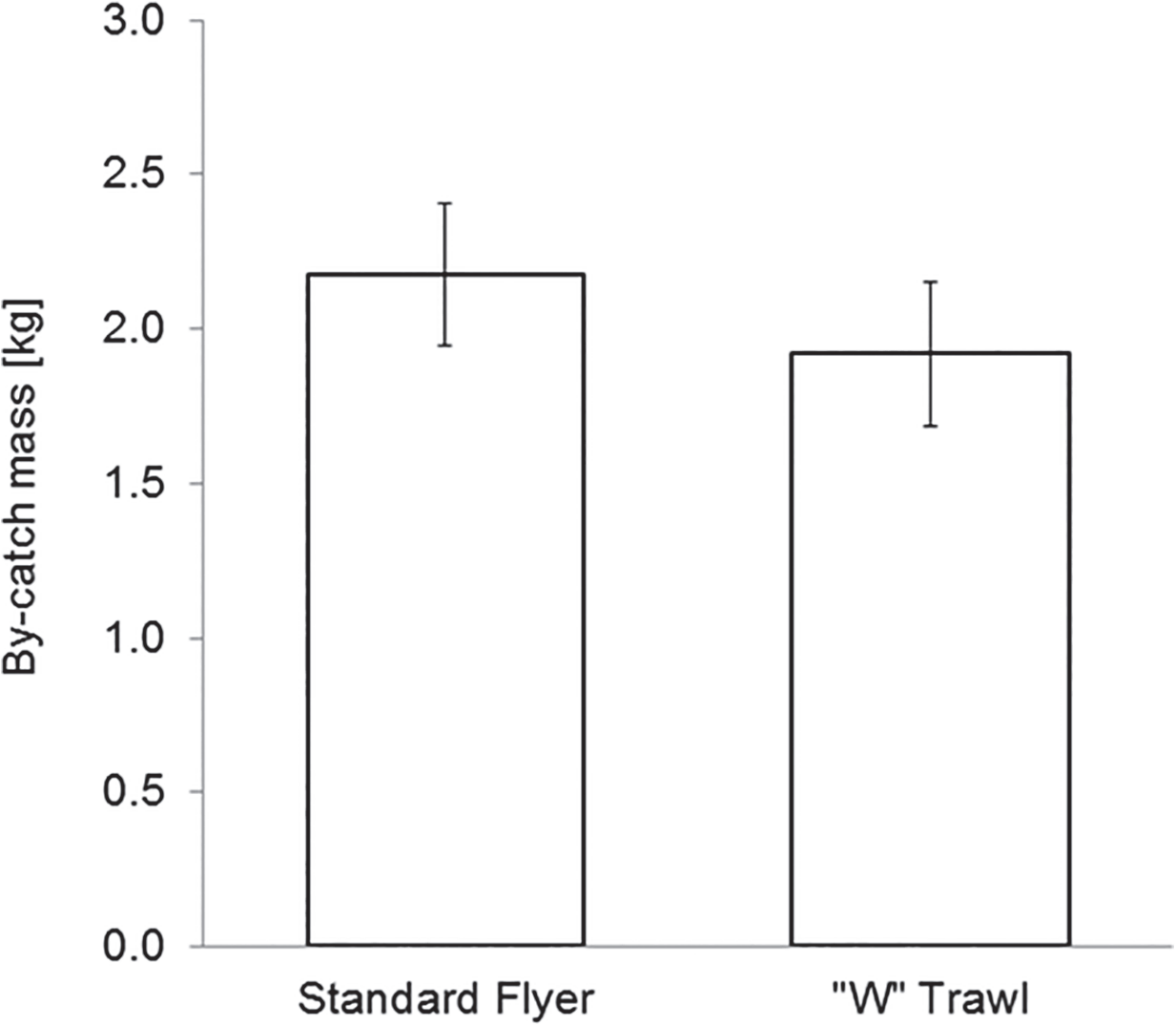
Predicted mean by-catch weight per tow, with 95% confidence intervals.

Fig. 12 shows the predicted mean catch for Tiger and Bay prawns. The standard trawl caught on average 4% more Tiger and Bay prawns by number per tow, while the ‘W’ trawl caught a slightly higher amount of prawns by weight—these differences, however, were not statistically significant (p < 0.05). Fig. 13 shows the predicted mean by-catch; whilst the standard trawl caught a 12% larger mass of untargeted species, the difference was not statistically significant (p < 0.05).

## Discussion

There was reasonable agreement between flume tank and field trial data in regards to the estimated standardised drag benefit of the ‘W’ trawl concept; being 14.5 ± 0.51% and 20.0 ± 0.7% respectively. The result could be stronger than expected in the field because both trawl systems were operating at spread ratios a few percentage points higher than that estimated as optimal from the flume tank work. Given the spread ratios actually achieved in the field, the flume tank results predict the drag benefit would be 15.2 ± 0.57%, which is still somewhat less than that indicated by the field data.

In absolute terms, the drag measurements of the trawl systems in the field were substantially higher than the predictions from the flume tank. This is likely due to the following reasons: (1) the flume tank estimations did not include ground chain forces or the effect of bridle in-pull forces, (2) drag due to field-material caught in the netting, (3) drag due to the catch in the cod-end, and (4) drag of lazy lines (used for cod-end retrieval) and towing wires. In the ‘W’ trawl system, a significant portion of these drag additions will have been redistributed onto the centre line tongues, and hence the relative drag difference between the two systems is similar between the flume tank and field data.

A factor that could cause both higher drag, in absolute terms, and greater drag benefit for the ‘W’ trawl in the field, is greater drag than expected from the otter boards. It could be that the L/D for the otter boards in the field was considerably less than the value of 1assumed during the flume tank work, which is possible given the muddy seafloor characteristics of Moreton Bay.

Drag implications of alternatively using diamond (T0) and square (T45) mesh in the principle parts of simple models of conventional and ‘W’ trawls were previously established [6]. One of the outcomes of that work was an estimate of the drag benefit of the ‘W’ trawl system compared to conventional systems. For the optimal ‘W’ trawl devised at that time the drag benefit was estimated to be ∼8%, but it was hypothesised that the drag benefit in a commercial context would likely be higher. The models used in that study were specially designed to equalise the proportion of trawl netting contained in the side sections compared to upper/lower panels, and a significant implication of this was that the trawls had very steep side tapers and generally very little twine area. For this reason it was hypothesised the drag benefit of the ‘W’ trawl compared to the conventional Florida flyer was subdued because the implementation of the upper and lower tongues generated a large relative drag penalty. In the present study, commercial style trawls were used and as expected the practical drag benefit of the ‘W’ trawl system is indicated to be significantly higher than that original estimate.

Standardised fuel usage of 2.88, 2.44 and 2.2 litre of fuel per hectare (swept area) was reported for typical Florida flyer trawls in single, twin and quad rig configurations respectively [5]. Based on these results, quad rig has a relative standardised drag of 0.906 compared to the twin rig. Table 4 shows that a twin configuration of ‘W’ trawls has a relative standardised drag of 0.800 compared to the standard twin rig. Therefore, twin ‘W’ trawls can be expected to produce a relative drag of 0.88 compared to traditional quad rig (refer to (d) and (e) in Fig. 1). The superior engineering performance of twin ‘W’ trawls over quad rig can be hypothesised to be due to the fact that the twin ‘W’ trawl system has half the number of side netting panels, which have a high drag contribution compared to the extra netting in the upper and lower sheets of the ‘W’ trawl.

As expected, there were low prawn catch rates for both trawls: firstly, the tested nets are not full size commercial gear, and they are used to locate areas of high abundance of target species; and secondly, the experiment was conducted out of season. Despite there being a small predicted reduction in the number of prawns caught for the ‘W’ trawl, because it was not statistically significant we do not envisage the new design to have inferior catching performance. This however warrants further investigation.

## Conclusions

Through the presented work, an innovative prawn trawl design was developed and a practical drag reduction has been demonstrated from testing in the flume tank and in the field. The specific findings and their implications are:

- 64% of trawl drag can be effectively redirected from the wings to the centre line in a small commercial T0 ‘W’ trawl with bracing ropes at a hanging ratio E = 0.707 installed down the centre lines of the upper and lower panels.
- Both flume tank and field data showed the use of ‘W’ trawls in a single rig or twin rig configuration produces significant standardised drag benefits. In the field the drag benefit was ∼20% compared to the use of standard trawls. Additionally, ‘W’ trawls in twin rig is expected to provide ∼12% drag savings compared to quad rig with standard trawls, and a practical advantage of having a reduced number of cod-ends, By-catch Reduction Devices and Turtle Exclusion Devices.
- Analysis of the catch data showed no statistically significant difference between the conventional and ‘W’ trawls. Further and more extensive sea trials are recommended with full size commercial trawls to establish comprehensively the catch-per-unit of fuel benefits of ‘W’ trawl systems.

## Supporting Information

S1 Dataset (XLSX)
